# Mechanisms of Stigmatization in Family-Based Prevention and Treatment of Childhood Overweight and Obesity

**DOI:** 10.3390/children10101590

**Published:** 2023-09-23

**Authors:** Didde Hoeeg, Katherine L. Frohlich, Ulla Christensen, Dan Grabowski

**Affiliations:** 1 Department of Health Promotion, Steno Diabetes Centre Copenhagen, Borgmester Ib Juuls Vej 83, 2730 Herlev, Denmark; dan.grabowski@regionh.dk; 2 École de Santé Publique & CRESP, Université de Montréal, 7101 Avenue du Parc, Montréal, QC H3C 3J7, Canada; katherine.frohlich@umontreal.ca; 3 Section of Social Medicine, Department of Public Health, University of Copenhagen, Gothersgade 160, 1123 Copenhagen, Denmark; ulch@sund.ku.dk

**Keywords:** childhood obesity, overweight, treatment, prevention, stigmatization, CMO configuration

## Abstract

It is well established that overweight and obesity are often accompanied by stigmatization. However, the influence of stigmatization on interventions for overweight and obesity remains unknown. Stigma may be particularly harmful to children. This study aimed to examine how stigmatization affects efforts to reduce childhood overweight and obesity through family interventions. This research was conducted in a socially disadvantaged area in Denmark. Twenty-seven families and forty professionals participated in in-depth interviews or workshops. The data were analyzed using CMO configurations from a realist evaluation and the theory of stigmatization developed by Link and Phelan. Thus, an abductive approach was employed in the analysis, with its foundation rooted in the empirical data. The study found that the mechanisms of stigmatization could 1. restrain professionals and parents from approaching the problem—thereby challenging family recruitment; 2. prevent parents from working with their children to avoid eating unhealthy food for fear of labeling the child as overweight or obese; and 3. cause children with obesity to experience a separation from other slimmer family members, leading at times to status loss, discrimination, and self-stigmatization. The study showed how the mechanisms of stigmatization may obstruct prevention and treatment of childhood obesity through family interventions. It is suggested that the concept of stigma should be incorporated into the program theories of interventions meant to reduce childhood overweight and obesity.

## 1. Introduction

Childhood overweight and obesity have increased during recent decades and constitute a major problem for public health worldwide [1]. However, no country has successfully reversed its obesity epidemic, as the systemic and institutional drivers of obesity remain largely unchanged [2]. Overweight and obesity are caused by a complex interplay between genetic, behavioral, cultural, environmental, and economic factors, yet most interventions are focused on individual responsibility, disregarding social determinants [3,4,5]. Social inequality in childhood overweight and obesity is well documented, with the highest rates among children in deprived areas of Western countries [6,7,8].

The complex links between low socioeconomic status (SES) and unhealthy lifestyles in socially vulnerable groups stem from multiple factors. Low SES families often struggle with financial constraints, restraining their access to nutritious food and fitness facilities. Furthermore, economic instability and limited opportunities can elevate chronic stress levels, fostering unhealthy coping methods, such as overeating and resistance to habit changes. Vulnerable families often reside in areas with many fast-food shops, inviting unhealthy eating habits and discouraging physical activity due to lack of parks and safe recreation spaces. Additionally, limited access to quality education and health information among low SES parents can result in a lack of knowledge about healthy choices and difficulties in comprehending complex health advice. These factors illuminate why traditional behavior change interventions frequently fall short when targeting low SES families [2,4,5].

Childhood overweight and obesity are linked to physical and psychological health complications, such as type 2 diabetes, hypertension, and reduced liver function [9]. In addition, overweight and obesity are highly stigmatized in Western society [10]. The Lancet Commission (2019) highlighted the pervasive challenges of weight stigma, which results in individuals with obesity being blamed and prejudged as unintelligent, ugly, incompetent, lazy, and lacking self-control [2]. Children and adolescents with overweight or obesity are also at risk of being stigmatized by peers, teachers, parents, and family members [11,12,13]. However, despite increased research on weight stigma, there is still limited knowledge about weight stigma in relation to interventions and pediatric healthcare settings [14].

It has been suggested that interventions should evaluate for stigma, and researchers should systematically account for the influence of weight stigma when studying overweight and obesity [10,15]. It is necessary to understand the goal and the mechanisms of stigmatization in order to diminish it [16,17]. In general, stigmatization applies to behavior perceived as voluntary to increase conformity with norms [18]. Thus, weight-stigmatization derives from the public understanding that individuals can control their own weight and are responsible for doing so [17,19]. This understanding is evident in most interventions to prevent or treat overweight and obesity among children.

Often, interventions are family-based and include physical activity and dietary components [20,21]. However, such interventions face challenges, including recruitment and retention of families, and have relatively small effects [20,21,22]. While RCTs have shown positive effects, translating and scaling up these interventions into real-world settings may not produce the same outcomes [23]. The context in which an intervention is introduced plays a crucial role in determining its success [24,25]. Therefore, Moore and Evans (2017) argued for a better understanding of the system in which an intervention is implemented, rather than viewing interventions as isolated packages of components described in isolation from their context [26].

Pawson and Tilley (2004) argued that a key requirement is to give consideration to the different layers of social reality that make up and surround programs. In their writing on realist evaluation, they furthermore stress four linked concepts for explaining and understanding interventions: ‘context’, ‘mechanisms’, ‘outcome’, and ‘context–mechanism–outcome pattern (CMO) configuration’ [24,27]. Context describes those features in which interventions are introduced that are relevant to the operation of the intervention mechanisms [24,28]. Mechanisms are the (often hidden) responses programs activate among stakeholders and participants, which result in an outcome [27]. Thus, the aim of the CMO configuration is to understand how mechanisms emerge in a specific context and how they affect the given intervention.

To help distinguish between relevant context and mechanism, Dalkin et al. (2015) suggested an alternative extension and operationalization of the CMO formula. The new formula (M (Resource) + C → M (Reasoning) = O) highlights the fact that resources must be introduced into a pre-existing context, which collaborate in generating an individual’s reasoning, leading to an outcome. Distinguishing the resources introduced into contexts from the ‘reasoning’ this generates can provide both an operational and a conceptual clarification of the mechanisms involved. Dalkin et al. (2015) argued that this distinction can enable researchers to clearly understand the role of context in activating mechanisms, thus helping to develop their explanation of how interventions work. In this current study, ‘resources’ are operationalized as ‘intervention activities’ which can be initiated and performed by both parents and professionals. Additionally, a slightly different approach to Dalkin et al.’s (2015) framework was applied, with a focus on the ‘intermediate outcome’ [29], which encompasses factors that could mediate the potential health benefits of the intervention activities.

Moore and Evans have emphasized the use of theory to comprehend participants’ reasoning [26]. Given that the analysis adheres to the principles of realistic evaluation [24], theory plays a vital role in elucidating the identified mechanisms. Therefore, the analysis incorporates Link and Phelan’s (2001) theory of stigmatization to unveil the mechanisms of stigmatization identified in the data. This theory was chosen due to its process-oriented understanding of stigma, which provides valuable explanatory strength to the analysis of how mechanisms of stigmatization emerged in the specific context and how they affected family-based initiatives to reduce childhood overweight and obesity.

In the following, the stigma theory defined by Link and Phelan (2001) will be described. The stigma theory will be used in the theoretically based reasoning within the CMO configurations. The CMO configurations also consist of the pre-existing contextual features relevant to the operation of intervention mechanisms [24]. In the section ‘Context and Setting’, the pre-existing context of relevance for the study will be presented. In the analysis, the reasoning of professionals and parents will be unfolded using the stigma theory before finally presenting three different CMOs in the discussion section.

## 2. The Theory of Stigmatization

Link and Phelan defined stigma as the co-occurrence of four main components and conceived of it as a social process occurring between people that is contingent on the use of power [30]. According to Link and Phelan, the four components of stigma include when people distinguish and label human differences (labeling); when dominant cultural beliefs link labeled persons to undesirable characteristics (stereotyping); when labeled persons are placed in distinct categories to separate “us” from “them” (separation); and when labeled persons experience status loss and discrimination leading to unequal outcomes (status loss and discrimination). Stigma is a matter of degree—not all four components need to appear to the same degree in the social process of stigmatization. Lastly, Link and Phelan described the socio-psychological process of stigma that operates though stigmatized persons, which they understood as embedded self-stigma, where stigmatized people expect to be discriminated against, even before an act of discrimination has occurred. People belonging to stigmatized groups expect that other people will devalue and discriminate against them, which leads stigmatized people to devalue themselves [30].

The objective of the present study was to investigate the potential mechanisms of stigmatization generated when attempts to reduce childhood overweight and obesity were made within the social context of a socially disadvantaged area. Dalkin’s (2015) operationalization of the CMO formula was used as an analytical tool to study the mechanisms of stigmatization (as reasoning) activated in the social context by an ‘intervention activity’. The study examines three separate intervention activities one by one to reinforce the focus on relevant contextual features and the stigma mechanisms activated.

## 3. Methods and Participants

The present study was based on three years of research conducted in a rural, disadvantaged part of Denmark. The research focused on two things: designing a family-based intervention to prevent overweight among children 2–6 years of age and evaluating a family-based intervention to treat childhood obesity among children 9–12 years of age [31,32]. The current study (including data from both interventions) contained the families’ and professionals’ perspectives on family-based prevention and treatment of childhood overweight and obesity. The study involved 27 families and 40 professionals, and the data were collected in three steps.

To gain knowledge about family life prior to the intervention in relation to the prevention of childhood overweight and obesity, four family workshops were conducted involving 12 families whose children had emerging obesity and were between 2 and 6 years of age (For more details on methods, participants and ethics see Hoeeg et al. 2020a [32]). Workshops as a research method have the potential to create new insights and self-reflections among the participants through peer-to-peer discussions [33,34]. Each family workshop lasted 2–3 h and took place at a local day-care institution and concluded with all participants eating dinner together. Siblings were also invited to enable both parents to participate in the workshop. Every family workshop began with involving parents and children together. Afterwards, parents and children were spilt up in two separate rooms, which created a child-free space where the parents could elaborate on their thoughts without the presence of their children.

To gain knowledge about family life in relation to the treatment of childhood obesity, a total of 15 interviews were conducted with families whose child was between 9 and 12 years of age and enrolled in a family-based intervention to treat childhood obesity (for more details on methods, participants and ethics see Hoeeg et al. 2020b [31]). The family interview was considered an appropriate method to gain insight into the families’ dynamics and everyday life while still taking ethical precautions on the involvement of children into account [35,36]. The interviews and workshops were focused on family life in relation to healthy living prior to the intervention as well as experiences with the intervention in families’ everyday lives.

To gain knowledge about the professionals’ perspectives on the reduction in childhood overweight and obesity, six workshops were conducted involving 40 professionals (including day-care professionals, health nurses, health consultants, professionals from the pediatric out-patient clinic, and sports and leisure practitioners) The workshop topics included the barriers that kept families from living healthier lives as well as the professionals’ ability to address weight issues in families. Characteristics of all participants are presented in Table 1. The parents’ occupations were recoded into social classes in accordance with the HBSC categorization scheme [37] (see Table 2).

All professionals and families gave their informed written consent before the workshop or interview began. The consent form included enough lines so that each participating family member could sign it individually—including the children. All interviews and workshops were transcribed verbatim, while sensitive characteristics were anonymized or recoded to ensure anonymity.

## 4. Analysis

The analysis was inspired by the principles of thematic analysis described by Braun and Clarke (2006). First, all the transcripts were read and the initial codes generated. During this first step, it was apparent how dominant stigma was throughout the data. Subsequently, the analysis moved back and forth between Link and Phelan’s theory and the data to recode data extractions on stigma. Following that, a search for overall themes related to stigma was undertaken throughout the dataset. Subsequently, inspired by the CMO configuration, suggested by Dalkin et al. (2015), components of stigma were identified to illustrate the participants’ reasoning in response to an ‘intervention activity’ (activities performed by professionals or parents). Themes were then defined and refined, and quotes were selected to illustrate finding [38]. The participants’ reasoning is presented in the results section, while the CMO configurations are presented in the discussion. In this manner, an abductive approach was employed in the analysis, with its foundation rooted in the empirical data.

## 5. Context and Setting

The study was conducted in a Danish municipality with high rates of overweight and obesity among adults as well as children compared with the average Danish rates [8,39]. In Denmark, municipalities are responsible for the prevention of overweight, while pediatric outpatient clinics are responsible for the treatment of childhood obesity [40]. Furthermore, the municipality is characterized as a disadvantaged area as it has a higher-than-average rate of citizens who depend on social benefits and of children living in poverty. It has a low average life expectancy as well as a low average disposable income compared with the average levels across Danish municipalities [41]. In Denmark, professionals working with children and in healthcare are required by law to report any child welfare concerns. This may lead to social interventions in families.

Local governments have the responsibility and legal authority to potentially remove a child from her/his parental home if they are concerned about the child’s welfare and if the parents are considered unable to take proper care of their child [42]. This particular geographical area of Denmark has a high level of vulnerable children (5.6%) and, correspondingly, has a relatively high number of cases where children are removed from their parental home due to child neglect (1.9%) compared to other areas of Denmark [43]. Hence, in the present study, the relevant features of the context of the ‘intervention activities’ under study are the divided organizational responsibility of prevention and treatment in Denmark; the sociodemographic and health characteristics of the intervention area; a relatively high occurrence of children in care; and lastly, the overall stigmatization linked to overweight and obesity, as described in the background section [15].

## 6. Results

### 6.1. A Touchy Subject—Approaching Families

Several participating families had previously been in contact with municipal authorities for different reasons. Some families expressed having too many professionals involved in their family life—describing themselves as being a ‘case’ in the system. This was a general concern among some families.

*“We have a family consultant (…). And we also have a so-called health nurse—Now we have chosen to say that when he [son] returns home from the weight loss camp, it is the nurses (Children’s outpatient clinic] who run it and not the health nurse (municipality), because we don’t want more people involved”. (Mother, interview)*

*“I have experienced one mother, who was like ‘her child should definitely not be enrolled in this’ because she immediately thought; no, my child should not be a case within the system”. (Professional, workshop)*

One family said that they had dropped out of an ongoing research project because too many professionals had become involved in their family life and they did not feel that they could cope with the research project as well. One professional also told us about an attempt to force a family to participate in the (voluntary) research project by expressing that the alternative would be a notification of their child’s health status to the municipality. Another family experienced a professional reporting their child’s weight status to the local municipal authorities.

*“It all started with you (son) visiting the health nurse at school. And then she wanted to put him on the scales—or… she started by sending notifications to the municipality that he had gained so and so many kilos within a year”. (Mother, interview)*

Throughout the data, the general power difference between those with overweight or obesity and those without was obvious. Some families conveyed that they had sensed there was an ongoing ‘hunt’ for those with overweight or obesity.

*“(…) Actually ‘healthy living’ has (…) always been imposed on overweight people. Many overweight people have been hounded with; ‘get healthy now’, ‘eat right now’, ‘do it now’, (…), so it has really been made into a bit of a smear campaign (…)”. (Father, workshop)*

This was also experienced indirectly by some of the children. One mother related how her son felt that his teachers monitored his lunch habits but not those of other classmates.

*“In relation to the milk-slices you can buy and petit yoghurts, with massive amounts of sugar in them. (…) He (son) said himself, that there are other children bringing these for lunch in the class. Why haven’t they mentioned that? Why is it just me? (…)”. (Mother, interview)*

The power difference was also experienced among the professionals. Some professionals conveyed that the weight issue was such a sensitive topic that they felt uncomfortable approaching families to offer them help and support. It was evident that the professionals knew they had a responsibility to support families that had issues with childhood overweight or obesity, but they felt unsure as to how to approach families and interfere in their lifestyle.

*“It requires that you break the taboo and dare to say it”. (Professional, workshop)*

The professionals struggled with the families that were reluctant to deal with the overweight and obesity issue, while some of the parents, paradoxically, felt that the professionals were reluctant to deal with it.

### 6.2. A Hurtful Label—Telling Children about the Obesity Issue

There was a clear concern among parents regarding how and when they should tell their children about their overweight. They were concerned about how old children should be when they are told—or whether they should be told at all. They also found it difficult to restrict their children’s food intake without telling them why they had to impose the restrictions. These parents found it challenging to prevent their children from consuming too much unhealthy food—in particular in social situations, e.g., birthdays or family visits.

*“It’s hard to tell her (daughter) “no, you’re not allowed to have that Halloween cake, because…?”—what do you say then? I think that’s been the hardest part”. (Mother, workshop).*

*“(…) The kids ask for it again and again, because the other children, who may not have these tendencies to gain weight, are allowed. “Why are we not allowed to go to McDonalds?”… and then tell a 6-year-old “Honey, it’s because you’re…?”. (Father, workshop)*

These parents feared that if their children were labeled as overweight or obese, they would experience discrimination. Often this expectation was based on the parents’ own experiences.

*“I struggled with overweight my entire childhood and I was bullied because of it. So, I hope my children don’t have to go through the same thing. (…) I don’t want them to experience what I’ve experienced”. (Mother, workshop)*

With the older children, several parents found it easier when a health professional told the child about the weight issue. Nevertheless, some parents also described health professionals as being too direct by calling the children “fat”, which made them doubt whether this was the best way to tell the children. Other parents trusted the health professionals to inform their children in the right way, even though it seemed harsh.

*“Because it’s really hard to tell your child that it’s also because you are… It can be tough. Then she (nurse) says it in a slightly harsher way and that’s probably also why he (son) sometimes gets a little upset. You already know, but it still sounds harsh”. (Mother, interview)*

When a health professional labeled a child as obese, it often made the child sad or angry, demonstrating that the label is highly undesirable.

*“I was quite mad because I was called fat” (Boy 12 years, interview).*

### 6.3. Obesity Stigma—An Unintended and Subtle Process

During the interviews or workshops with the families, parents reflected on the cause of their child’s weight status. When doing so, they often used common negative stereotyping linked to people with overweight or obesity (e.g., being sloppy, lazy, enjoying food too much, and lacking self-discipline, motivation, and personal control).

*“She (daughter) was not very old when we realized that she could consume huge amounts of food (…) And you (daughter) were also born lazy. Already as a baby, you were lazy”. (Mother, interview)*

*“She (daughter) can’t control it (food/snacks) anymore. It’s like she needs the fix”. (Mother, workshop).*

This indicated that the children, to some extent, had come to be identified with these stereotypes, stressing individual behavior as a cause of their weight issues. When families implemented the family obesity intervention, a ‘separation’ was often generated between the child dealing with obesity and the family members without weight issues.

*“It’s been tough from time to time. Especially when his older brother can eat it without gaining weight, while he (son) can’t and isn’t allowed to either”. (Father, interview)*

Even though several families *described* how they had attempted to involve the whole family in the intervention, this did not seem to happen in practice, as slim family members were often exempt from the new healthy efforts (often siblings or fathers). This meant that the children in the intervention often received little familial support to change their unhealthy habits into healthier ones, which made it difficult for those children to adhere to new healthy habits. Some of the children also experienced ‘status loss and direct discrimination’, in the form of being bullied by other family members (siblings) or peers.

*”My older brother called me a fat cow. He does this all the time, (…) when we’re playing and I accidentally kick him (…). Then he calls me a fat cow, because he knows I’m going there (Children’s outpatient clinic). (…) Sometimes I start to cry, when he says it—Because I am doing my best”. (Girl 9 years, interview)*

Some children did not want their parents to tell others they had been labeled obese and were enrolled in an intervention because they expected this would cause peers to discriminate against them.

*“You (daughter) haven’t been fond of telling others that you’re in the programme”. (Mother, interview).*

*“No. (…) Because I was afraid someone would tell some friends about it, (…) who would tell their brothers, for example. I think some brothers they bully a lot, (…) and I wouldn’t like that”. (Girl 9 years, interview)*

Some parents observed how the weight discrimination had affected their children’s well-being overall, and there were indirect signs that some children had begun performing aspects of ‘self-stigmatization’.

*“(…) It’s really frustrating. You don’t want your kid to be bullied, right? So of course, it affects you when you can see that the big one is having a hard time. What really hurts is when he comes home from school sad… Then he gets self-destructive again (…)”. (Father, workshop)*

## 7. Discussion

The paper commenced by highlighting the specific contextual features that are part of a CMO configuration. The theory of stigma was employed to aid in unraveling the reasoning of professionals, parents, and children. The aim was to elucidate certain barriers to family interventions in this area. Three distinct CMO configurations were employed.

First, it was found that professionals perceived weight issues to be so sensitive that they were restrained in how they approached the problem with parents, while some parents seemed concerned about becoming a ‘social case’ in the municipal system. The latter emphasized the power relation between the professionals and families, power being an essential component of the process of stigmatization. To analyze these findings through the lens of a CMO configuration, resources (‘intervention activity’) were conceived as the recruitment of families to interventions aimed at reducing childhood overweight and obesity. This recruitment was embedded in the context related to the professional’s strict duty to notify the authorities if they were concerned about a child’s welfare as well as to the local government’s authority to instigate a forced social intervention in families. The context was also related to the fact that overweight and obesity are highly stigmatizing.

The data showed the reasoning of professionals, revealing that they perceived the weight issue to be too sensitive to express in words. At the same time, parents’ reasoning revolved around the risk of becoming a ‘social case’ in the system. The intermediate outcome was that the professionals restrained themselves from interfering in families, while some families restrained themselves from seeking help from professionals. In this way, the underlying power in the social process of stigma became clear and, in the present case, it seemed to inhibit the recruitment of families to supportive interventions. It may be challenging for municipal professionals, who have the power to notify authorities that can initiate forced social interventions in families, to also be responsible for offering help and support to families, with a view to preventing childhood overweight and obesity. Even though children are not being removed from families due to obesity issues, the risk of the municipality forcing a social intervention in families is present and seems crucial, as some families felt persecuted by professionals due to weight issues. Thus, we showed how, for some vulnerable families, the professionals’ obligation to notify authorities became intertwined with issues of childhood overweight and obesity.

These current findings partly align with a study showing that professionals feared approaching the subject of weight with parents. The professionals believed that parents were reluctant to deal with or even talk about the issue of weight for fear of “labeling” their child [44]. Recruiting families to interventions on childhood overweight or obesity is known to be challenging—particularly recruiting socially vulnerable families [22]. The current study showed how the mechanisms of stigmatization seemed to be important when recruiting socially vulnerable families living in the disadvantaged area under investigation. In such cases, it is important to be careful not to further blame the families and inadvertently make children and parents of low socioeconomic status and with issues of overweight feel even more marginalized, disadvantaged, and hopeless [45].

Second, it was found that the parents of young children were so concerned that their children would be labeled as overweight or obese that it limited their ability to help their children avoid eating unhealthy food and snacks.

To analyze these findings, another CMO configuration was used, in which the parents’ attempts to alter the unhealthy habits of the child were conceived as the resources (‘intervention activity’), while the contextual feature here was the fact that being overweight or obese is a highly stigmatizing condition.

The data showed how the parents’ lines of reasoning were related to their concerns about labeling their young children as overweight or obese. The parents did not want to make their children feel deviant, thus rendering the disallowance of certain foods problematic. They feared that altering the children’s eating habits would involve labeling their children as overweight and obese, causing the child to feel bad about him/herself. This reasoning seemed to inhibit the parents’ ability to alter their young children’s eating habits as an ’intermediate outcome’, which illustrated how mechanisms of stigmatization seem to hinder obesity-preventive parenting. However, the parents of older children were often pleased that a health professional had informed the child about the weight issue, even though it sounded harsh.

In line with this, a previous study found that parents tried to hide the fact that their young children needed to lose weight in an effort to protect them from feeling stigmatized. However, to encourage their children to take on more responsibility, these parents became more forthright about weight loss as the children grew older [46]. Another study showed how parents had come to stereotype their children, thus making the children feel ashamed, angry, and sad as well as ashamed for not being able to lose weight [47]. The results of these qualitative studies are in line with the findings on the role of stigma within families enrolled in an obesity treatment intervention.

Additionally, there was evidence of parents stereotyping their child, which seemed to generate a separation between the child with obesity and the family members without weight issues. This separation was made in an effort to specify who needed to adopt healthier habits. Further, the data revealed how some children had experienced status loss and discrimination among siblings or peers, which in some cases had caused the children to perform aspects of self-stigmatization.

To analyze these findings through the lens of a third CMO configuration, the parents’ implementation of a family intervention to treat childhood obesity was conceived as the resources (‘intervention activity’). The contextual feature here was the fact that being overweight or obese is a highly stigmatizing condition. The data showed how parents seemed to unknowingly reason their implementation approach by ‘stereotyping’ the reason for the child’s weight issue and further by employing a ‘separation’ between the child with obesity and other family members without weight issues. Combined with the fact that some children experienced ‘discrimination and status loss’, either at home or at school, this shows how several of the components of stigma had emerged among these children. This seemed to have caused some children to devalue themselves and perform self-stigma as an intermediate outcome. It is well known that stigma and self-stigma are counter-productive with regard to losing weight as well as improving health and well-being [48,49]. Thus, the overall outcome of these findings is that the mechanisms of stigmatization seemed to inhibit intervention activities aimed at preventing or treating childhood overweight and obesity through family interventions. These findings are in line with earlier research [47,50,51,52,53]. 

Stigma is a latent contextual feature, and intervention activities can either activate or amplify the mechanisms of stigmatization. The findings showing that the mechanisms of stigmatization can obstruct preventive intervention activities and that stigma can unintentionally be reinforced through family interventions to treat childhood obesity are critical. The literature has rarely addressed the fact that well-intentioned interventions may result in unintended adverse effects that harm participants [54]. Likewise, the role of stigma has rarely been considered when obesity interventions are designed [10]. The knowledge that interventions to treat childhood obesity may run the risk of doing more harm than good among the enrolled children constitutes a major ethical concern [45]. Thus, the current study concludes that stigmatization is a pivotal mechanism influencing the work to reduce childhood overweight and obesity through family interventions.

## 8. Strength and Limitations

Utilizing CMO configurations and theory-based reasoning and drawing inspiration from Link and Phelan’s (2001) stigma theory proved to be a productive approach in illustrating how the mechanisms of stigmatization as a process can impact efforts to prevent or treat childhood overweight and obesity. A limitation of the study is that stigmatization was not the primary focus but emerged during data analysis. Additionally, as all data were collected through family interviews or workshops, the presence of other family members may have limited candid responses about weight stigma experiences. The researchers were aware of this risk and tried to encourage all family members to convey their experiences and perspectives. The format of the interviews or workshops prompted reflections among both children and adults—and also among those hesitant to speak in front of others.

## 9. Implications

When interventions to reduce childhood overweight and obesity are designed, the current study suggests that stigma should be considered in the intervention design. Incorporating stigma into program theories, as a non-intended mechanism, can raise awareness of how it affects interventions (from recruitment and implementation to families’ internal management of an obesity intervention). This, in turn, will bring focus to the ways in which stigma can be approached and hopefully minimized. Further, the findings and theoretical approach may also be applicable to other behavioral interventions that can be stigmatizing for participants.

## Figures and Tables

**Table 1:** Characteristics of participants.

Professionals in Workshops		Number Participants
Day-care professionals		15
Health nurses		13
Health consultants		6
Professionals from the pediatric out-patient clinic		2
Sports and leisure practitioners		4
**Total**		**40**
**Family workshops**	**Number participants**	**Total**
Families		**12**
Parents		22
Mother	12	
Father	10	
Children (0–11 years old)		23
**Family members in workshops**		**45**
**Family interviews**	**Number participants**	**Total**
Families		**15**
Parents		21
Mother	14	
Father	7	
Children (9–12 years old)		15
Female	10	
Male	5	
Age		
9 years	6	
10 years	4	
11 years	4	
12 years	1	
**Family members in interviews**		**36**
**Families in total**		**27**

**Table 2:** Parental social class *.

	Family Workshops		Family Interviews	
Social Class	Number of		Number of	
	Mothers	Fathers	Mothers	Fathers
I	0	0	0	0
II	0	0	0	0
III	4	0	3	0
IV	2	4	3	3
V	3	5	4	2
VI	3	1	4	1
Missing	0	0	0	1
Total	12	10	14	7

* Social class was coded according to HBSC. Code I is defined as a professional occupation with high SES. Code V is defined as an unskilled occupation with low SES, and Code VI is defined as economically inactive (e.g., student, unemployed, or ill) [37].

## Data Availability

Data is unavailable due to ethical restrictions.
